# Recyclable Organocatalyzed Poly(Thiourethane) Covalent Adaptable Networks

**DOI:** 10.3390/polym12122913

**Published:** 2020-12-04

**Authors:** Francesco Gamardella, Sara Muñoz, Silvia De la Flor, Xavier Ramis, Angels Serra

**Affiliations:** 1 Department of Analytical and Organic Chemistry, Universitat Rovira i Virgili, C/ Marcel·lí Domingo 1, Building. N4., 43007 Tarragona, Spain; francesco.gamardella@urv.cat; 2 Department of Mechanical Engineering, Universitat Rovira i Virgili, Av. Països Catalans, 26, 43007 Tarragona, Spain; sara.munozg@estudiants.urv.cat (S.M.); silvia.delaflor@urv.cat (S.D.l.F.); 3 Thermodynamics Laboratory, ETSEIB Universitat Politècnica de Catalunya, Av. Diagonal, 08028 Barcelona, Spain; xavier.ramis@upc.edu

**Keywords:** vitrimers, poly(thiourethane), thermosets, recyclability, covalent adaptable networks, organocatalyst

## Abstract

A new type of tetraphenylborate salts derived from highly basic and nucleophilic amines, namely 1,5-diazabicyclo[4.3.0]non-5-ene (DBN), 1,8-diazabicyclo(5.4.0)undec-7-ene (DBU) and triazabicyclodecene (TBD), was applied to the preparation of networked poly(thiourethane)s (PTUs), which showed a vitrimer-like behavior, with higher stress-relaxation rates than PTUs prepared by using dibutyl thin dilaurate (DBTDL) as the catalyst. The use of these salts, which release the amines when heated, instead of the pure amines, allows the formulation to be easily manipulated to prepare any type of samples. The materials prepared from stoichiometric mixtures of hexamethylene diisocyanate (HDI), trithiol (S3) and with a 10% of molar excess of isocyanate or thiol were characterized by FTIR, thermomechanical analysis, thermogravimetry, stress-relaxation tests and tensile tests, thus obtaining a complete thermal and mechanical characterization of the materials. The recycled materials obtained by grinding the original PTUs and hot-pressing the small pieces in the optimized time and temperature conditions were fully characterized by mechanical, thermomechanical and FTIR studies. This allowed us to confirm their recyclability, without appreciable changes in the network structure and performance. From several observations, the dissociative interchange trans-thiocarbamoylation mechanism was evidenced as the main responsible of the topological rearrangements at high temperature, resulting in a vitrimeric-like behavior.

## 1. Introduction

Thermosetting polymers, thanks to their permanent covalent bonds in the network structure, show outstanding mechanical and thermal properties. However, their permanent three-dimensional structure hindered this material to be melted or dissolved in any solvents, making it impossible to reprocess and recycle them. In view of the increasing amount of plastic generated, the recycling of this type of polymers is extremely important to prevent them from ending up in landfills and contaminating the environment. The development of covalent adaptable networks (CANs), covalently crosslinked polymers with the ability to be reshaped, to flow and to self-repair, represents a promising approach to improve the lifetime and recyclability of the thermosetting polymers [1,2,3,4].

Recently, networked poly(thiourethane)s (PTUs) have been reported as a new class of CANs thanks to the presence of thiourethane groups that can undergo exchange reactions at moderate temperatures [5,6,7,8]. PTUs are formed by the very efficient coupling reaction between thiols and isocyanate, which, in the presence of a base catalyst, can be considered a *click*-type reaction. The reaction is very fast without the formation of by-products and can be performed in air atmosphere, without the use of solvents. The resulting network structure presents high homogeneity and transparency, with a high refractive index, thanks to the presence of sulfur in the network structure [9,10]. The abovementioned characteristics, together with their good mechanical properties and easy preparation, make them interesting for many advanced applications and therefore worthy of being further investigated, representing an interesting alternative to the more common poly(urethane)s.

Poly(urethane)s (PUs) have already been reported to experiment network reconfiguration by trans-carbamoylation [11,12]. Poly(thiourethane)s are the sulfur analogues of poly(urethane)s, and thus, they can behave in a similar manner by a trans-thiocarbamoylation process. Moreover, since thiols are more acidic than alcohols and sulfide anions are more nucleophilic than alkoxides and better leaving groups, some positive kinetic effects in the bond interchange of poly(thiourethane)s could be foreseen. In two previous papers from our research group [6,7], we proved the existence of a reversible thiocarbamate exchange reaction in PTUs and the possibility to recycle them. In both studies, a tin-catalyst was used and the influence of the amount of the catalyst on the relaxation rate was demonstrated. When comparing the relaxation behavior of poly(thiourethane) and poly(urethane) networks, it can be observed that the activation energy, E_a_, is significantly lower for the former (between 70 and 100 kJ/mol) [6,7] than for the latter (around 140 kJ/mol) [13], which confirms the kinetic effect produced by the substitution of oxygen by sulfur.

In an excellent paper from Hillmeyer’s group, a mechanistic study of the exchange mechanism in poly(urethane) networks in the presence of a Lewis acid, tin (II) octoate, was reported [14]. The urethane reversion to isocyanate and alcohol was demonstrated to be the main responsible of the exchange mechanism. For these materials, a dissociative mechanism was proposed, in absence of free hydroxyl groups in the network, although the relaxation studies showed a vitrimer-like behavior without any sudden decrease in viscosity, fitting an Arrhenius model. Similarly, in our studies, a vitrimeric behavior was observed in the case of poly(thiourethane) networks prepared with a tin-catalyst [6,7].

The preparation of PTU networks has been classically performed by the use of dibutyltin dilaurate (DBTDL) as the catalyst, since the use of bases such as amines leads to a too quick reaction [15,16]. However, the use of amines is quite convenient, because it is said to lead to a click-reaction that produces a more homogeneous network, without the formation of unexpected moieties [17,18]. To avoid these limitations, in a previous paper [19], we investigated the advantages of using a thermally triggered base generator (1-methylimidazolium tetraphenylborate, BGMI), an organocatalyst, which liberates the corresponding amine at a defined temperature, allowing a good temporary control of the thiol-isocyanate reaction. Organocatalysts are more environmentally favorable than a tin-catalyst from a toxicity and pollution point of view, being considered greener than metallic catalysts [20,21].

As the use of catalysts plays a key role also in the rate of dynamic covalent-bonds exchange, their appropriate selection could affect the mechanism and the kinetics of the exchange process, tuning the desired reconfiguration properties [22,23]. As already studied by our research group, PTUs relax faster at higher catalyst concentration of DBTDL [6].

Regarding the trans-thiocarbamoylation process, Torkelson et al. [5] described that thiourethane linkages can be rearranged through associative and dissociative reversible pathways, depending on the proportion of thiol in the formulation. These poly(thiourethane) elastomers were prepared from aromatic diisocyanates, in the presence of a strong base 1,8-diazabicyclo(5.4.0)undec-7-ene (DBU) (0.0005%) and a 3% of triphenylphosphine (PPh_3_). DBU was used to form the network, and PPh_3_ contributed significantly to the efficient reprocessing of the elastomer.

**Scheme 1:**
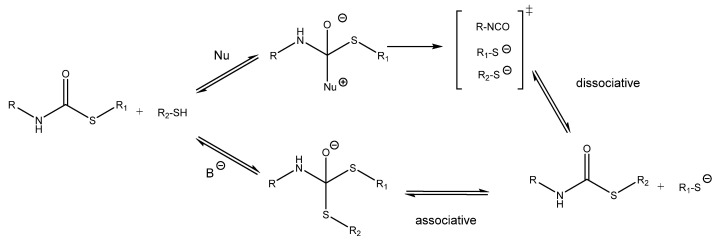
Associative-dissociative trans-thiocarbamoylation exchange mechanisms catalyzed by bases and nucleophiles, proposed by Bowman et al. [24].

Very recently, Bowman et al. [8] reported the recyclability of poly(thiourethane) elastomers by using three different type of catalysts: nucleophilic, basic and dual-role. The dual-role catalyst, 1,5-diazabicyclo[4.3.0]non-5-ene (DBN), leads to the most effective synergetic associative and dissociative thiourethane bond exchange mechanism. The dual associative-dissociative mechanism catalyzed by base-nucleophiles has been proposed by Bowman et al. [24] as depicted in Scheme 1.

In light of these considerations, the use of thermally generated base catalysts represents an interesting opportunity to reach a temporal control of the curing reaction, offering, at the same time, the possibility to increase the amount of catalyst, which could help to improve the rate of the exchange process. The use of such catalysts does not require any solvents, as it was reported by Torkelson [5] and Bowman [18].

In the present work, we explore and report the ability to undergo stress-relaxation of poly(thiourethane) networks prepared by using different tetraphenylborate salts of the following amines: 1-methylimidazole (1MI), 4-(dimethylamino)pyridine (DMAP), 1,8-diazabicyclo- (5.4.0)undec-7-ene (DBU), 1,5-diazabicyclo[4.3.0]non-5-ene (DBN) and triazabicyclodecene (TBD). Moreover, we investigate the relaxation behavior of these materials and their thermal behavior by thermogravimetry, to reach a good understanding of safe recycling conditions.

As in our previous study [6], 1,6-hexamethylene diisocyanate (HDI) and trimethylolpropane tris(3-mercaptopropionate) (S3) were selected as the monomers in both stoichiometric proportions and in excess of one of them. The virgin and recycled materials were characterized by thermomechanical and mechanical tests, which proved, together with FTIR analysis, that the recycling process can be performed if the experimental conditions are properly selected.

## 2. Materials and Methods

### 2.1. Materials

Trimethylolpropane tris(3-mercaptopropionate) (S3), hexamethylene diisocyanate (HDI), dibutyltin dilaurate (DBTDL), 1-methylimidazole (1MI) (pK_a_ = 7.1, N = 11.9) [25], triazabicyclodecene (TBD) (pK_a_ = 14.4, N = 16.16) [25] and sodium tetraphenylborate (NaBPh_4_) from Sigma-Aldrich (Saint Louis, MO, USA), were used as received. 4-(Dimethylamino)pyridine (DMAP) (pK_a_ = 9.7, N = 14.95) [26], 1,5-diazabicycle[4.3.0]non-5-ene (DBN) (pK_a_ = 13.7, N = 15.50) [8] and 1,8-diazabicyclo(5.4.0)undec-7-ene (DBU) (pK_a_ = 13.5, N = 15.29) [26] were supplied by Alpha Aesar (Kandel, Germany). Methanol (MeOH) and chloroform (CHCl_3_) were supplied by VWR (Darmstadt, Germany) and were used as received.

### 2.2. Preparation of Organocatalysts (BG)

Base generators (BGs) from different amines were synthesized by us, according to a reported methodology [27]. Firstly, 10 mmol of the selected amine was solubilized in 2.6 mL of water slightly acidified with 1 mL of conc., 36% HCl solution. Then, 11 mmol of NaBPh_4_ was solubilized in water and stirred until complete homogenization. The two aqueous solutions were mixed, and a white salt was formed as a precipitate. The salt was filtered, washed thoroughly with distilled water and MeOH, then recrystallized from a 4:1 mixture of MeOH and CHCl_3_, filtered and dried under mild temperature and vacuum. The purity of the synthesized compound was assessed via a differential scanning calorimetry (DSC) thermal scan, and its melting point was found to be similar to what is reported by other equivalent salts in literature [28,29,30]. The different base generators prepared where named as BGXXX, where XXX indicates the base used.

### 2.3. Preparation of the Formulations

To prepare the different formulations, the amount of base generator (BG) was conveniently selected to release 0.05% mol of the selected base. The BG of the selected base was first added to the thiol, and the system was kept under stirring, at 110 °C, for 45 min, until complete solubilization. The mixture was cooled down at room temperature, and then the required amount of diisocyanate was added, manually stirred and immediately poured into a mold or sent to analysis.

As an example, the formulations were composed by 1.90 g (11.29 mmol) of HDI, 3 g (7.53 mmol) of S3 and 0.77 mg (0.0094 mmol) of 1MI or 3.99 mg (0.0094 mmol) of BG1MI.

For the recycling and degradation studies, the thermosetting PTUs were also prepared with different stoichiometric ratios, and 0.1% mole of BGDBU was added as the catalyst. Typical amounts used are reported in Table 1.

### 2.4. Sample Preparation

For DMTA analysis, films were prepared by pouring the formulations on pre-silanized glasses and using Teflon spacers to ensure a homogeneous thickness of 0.5 mm. The formulations were cured at 60, 100 and 130 °C for 1 h at each temperature. The films were die-cut to obtain a rectangular specimen of 20 × 5 × 0.5 mm^3^ dimensions.

### 2.5. Stress-Relaxation Tests

Tensile stress-relaxation tests were conducted in a TA Instruments DMA Q800 analyzer (New Castle, DE, USA), using a film tension clamp on samples with the same dimensions as previously defined. To compare the rate of relaxation of the poly(thiourethane) vitrimers obtained by using different catalysts, a single stress-relaxation test was performed at 180 °C, for 1 h, at a constant strain of 1.5%.

To obtain the activation energy (*E_a_*) for each material, the sample was firstly equilibrated at 130 °C for 5 min, and a constant strain of 1.5% was applied, measuring the consequent stress level as function of time. After releasing the strain, the process was repeated by increasing by increments of 10 °C, until the final test temperature, 170 °C, was reached. The relaxation-stress σ(t) was normalized by the initial stress, σ_0_, and the relaxation times (*τ*) were determined as the time necessary to relax 0.37·σ_0_, i.e., (σ = 1/e·σ_0_). With the relaxation times obtained at each temperature, the activation energy values, *E_a_*, were calculated by using an Arrhenius-type equation:

(1) $$\ln\left(\tau \right)=\frac{E_{a}}{RT}-\ln A$$

where *τ* is the time needed to attain a given stress-relaxation value (0.37σ*_0_*), *A* is a pre-exponential factor and *R* is the gas constant. From the Arrhenius relation, the temperature of topology freezing (*T_v_*) was obtained as the temperature at which the material reaches a viscosity of 10^12^ Pa·s. Using Maxwell’s relation and *E’* determined from DMTA (assuming *E’* being relatively invariant in the rubbery state), τ* was determined to be around 10^5^ s in our systems. The Arrhenius relationship was then extrapolated to the corresponding value of τ*, to determine *T_v_* for each sample. To evaluate the thermomechanical properties of the different materials prepared, the evolution of tan δ and storage modulus with the temperature was investigated. A sample was tested in tension in the DMA Q800 analyzer, at a heating rate of 3 °C/min from 0 to 120 °C, with a frequency of 1 Hz and 0.1% of strain.

### 2.6. Thermal Degradation Studies

The thermal stability of the cured samples was studied by thermogravimetric analysis (TGA), using a Mettler TGA/SDTA 851e thermobalance (Columbus, OH, USA). All experiments were performed under inert atmosphere (N_2_ at 100 mL/min). Pieces of cured samples of 10–15 mg were degraded between 30 and 600 °C, at various heating rates, namely 1, 2, 5 and 10 °C/min.

### 2.7. FTIR Analysis

Fourier-transform infrared (FTIR) spectra were registered with a Bruker Vertex 70 (Billerica, MA, USA) in absorbance mode at a resolution of 4 cm^−1^ in the wavelength range of 400–4000 cm^−1^. The instrument is equipped with attenuated total reflection (ATR) accessory (Golden GateTM, Specac Ltd., Orpington, UK) which is temperature controlled (heated single-reflection diamond ATR crystal).

The evolution of the FTIR spectra of the poly(thiourethane) materials was followed isothermally at 180 °C in the ATR. The materials before and after being degraded in dynamic TGA, until 260 and 340 °C, were analyzed at room temperature. The spectra of original and recycled PTUs were also recorded at room temperature.

### 2.8. Gas Chromatography-Mass Spectrometry (GC-MS) Analyses

The stoichiometric PTU was degraded by heating 2 g of the material at 200 °C, for 1 h, in a sealed vial. The detection of the derived volatile products was performed in a HP6890 gas chromatograph and 5973 Mass selective detector (Agilent Technologies. Waldbronn, Germany), using an HP-5MS capillary column (30 m × 0.25 mm × 0.25 m) provided by Agilent.

### 2.9. Recycling

The recycled samples were obtained by cutting the crosslinked polymers and hot-pressing at 15 MPa into an aluminum mold, at 130 °C for 3 h. Recycled samples were die-cut in Type V from the new film obtained and were tested in tensile, under the same conditions.

Original and recycled samples were tested until break in tensile mode, at room temperature, using an electromechanical universal testing machine Shimadzu AGS-X (Shimadzu Co., Kyoto, Japan) with a 1000 N load cell at 5 mm/min and using Type V samples according to ASTM D638-14 standard. Three samples of each material were tested, and the average results are presented.

### 2.10. Dissolution Experiments

Dissolution experiments of crosslinked polymers were performed by the following procedure. Pieces of poly(thiourethane) samples of 0.1–0.2 g, which were weighed before the experiment, were placed into a vial. The vial was filled with 1,2-dichlorobenzene (DCB) or dimethyl sulfoxide (DMSO) closed and heated at 150 °C for 24 h, and then the vial was cooled down to room temperature. The polymer sample (if it still exists) was washed by dichloromethane and dried under reduced pressure, at 80 °C, overnight. After cooling down to room temperature, the sample was weighed, and the gel fraction was calculated.

### 2.11. Kinetic Analysis

From the dynamic experiments carried out by using a thermobalance, the mass loss of the sample is recorded. The degree of conversion is defined as follows:

(2) $$\alpha =\frac{m_{0}-m}{m_{0}-m_{\infty }}.$$

where *m* is the mass corresponding to a temperature *T*, *m*_0_ is the initial mass and *m*_∞_ is the mass of the substance at the end of the experiment.

In non-isothermal kinetics of heterogeneous condensed phase reactions, it is usually accepted that the reaction rate is given by Equation (3) [31,32].

(3) $$\frac{d\alpha }{dt}=\beta \frac{d\alpha }{dT}=A\;\exp\left(-\frac{E}{RT}\right)f(\alpha )$$

where *α* is the degree of conversion, *T* is temperature, *t* time, *f* (*α*) is the differential conversion function, *R* is the gas constant, *β* is the linear constant heating rate *β* = d*T*/d*t* and *A* and *E* are the pre-exponential factor and the activation energy given by the Arrhenius equation.

By integrating Equation (3), in non-isothermal conditions, the integral rate equation, so-called temperature integral, may be expressed as Equation (4).

(4) $$g\left(\alpha \right)=\int_{\;0}^{\;\alpha }\frac{d\alpha }{f\left(\alpha \right)}=\frac{A}{\beta }\int_{\;0}^{\;T}e^{-(E/RT)}dT$$

where *g* (*α*) is the integral conversion function.

By using the Coats-Redfern approximation to solve Equation (4) and considering that 2*RT*/*E* is much lower than 1, the Kissinger-Akahira-Sunose (KAS) equation (Equation (5)) may be written as follows [33,34,35]:

(5) $$\ln\left(\frac{\beta }{T^{2}}\right)=\ln\left[\frac{AR}{g\left(a\right)E}\right]-\frac{E}{RT}$$

For each conversion degree, the linear plot of ln(*β*/*T*^2^) versus *T*^−1^ enables *E* and ln [*AR/g*(*α*)*E*] to be determined from the slope and the intercept. Isoconversional kinetic parameters were obtained in this work by using Equation (5).

By integrating Equation (3) for isothermal experiments, we can obtain Equation (6):

(6) $$\ln t=\ln\left[\frac{g(\alpha )}{A}\right]+\frac{E}{RT}$$

This equation relates, for each conversion, the temperature and the time of degradation. The constant ln [*g*(*α*)/*A*]] is directly related to the value ln [*AR*/*g*(*α*)*E*] by *E/R*, which can be deduced from the non-isothermal adjustment (Equation (5)), if isothermal and non-isothermal curing take place under the same conditions.

In this work, we used ln [*AR*/*g*(*α*)*E*] and *E/R* obtained by dynamic experiments and Equations (5) and (6) to estimate the temperature at which the material can be recycled without significant thermal degradation.

## 3. Results and Discussion

### 3.1. Study of the Effect of the Catalyst on the Relaxation Behavior

**Scheme 2:**
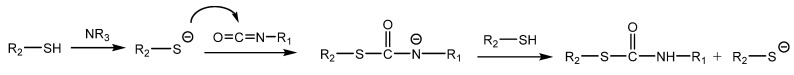
Proposed mechanisms of poly(thiouretane) formation in the presence of a basic catalyst.

The viscoelastic properties of poly(thiourethane) thermosets prepared from stoichiometric amounts of HDI and S3, in the presence of a base catalyst (1MI) and without using any solvent, were firstly investigated. In the presence of a base, the thiol-isocyanate reaction is very fast, and the system is difficult to process due to the low pK_a_ of thiol species and their easy deprotonation in presence of the base. Especially in the presence of a base with a high value of pK_a_, the reaction reaches the 100% of conversion in a few seconds, even when greatly reducing the amount of the catalyst. The mechanism of thiol-isocyanate reaction in presence of a base is proposed in Scheme 2.

**Scheme 3:**
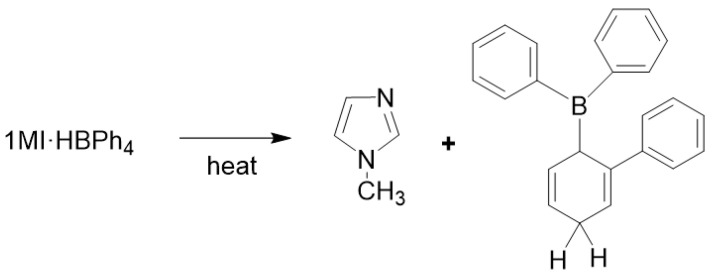
Thermal activation of the base generator BG1MI.

Based on the expertise of our research group on temporal and kinetic control of the curing processes, we chose as the catalyst a base generator that releases the base after the application of an external stimulus, as shown in Scheme 3.

The use of such latent bases could also allow us to increase the amount of catalyst in the formulation, which hypothetically could lead to an acceleration of the exchange mechanism.

The effect of using the BG on the thermal and rheological properties of the material was evaluated and compared with a similar system that was prepared by using the corresponding base catalyst. We selected as the base the one with lower pK_a_ and N values, and we prepared two samples of poly(thiourethane) from HDI and S3, one with 0.05% mole of 1MI and another with the same mole proportion of BG1MI.

The thermal behavior of the materials prepared with the same mol proportion of 1MI and BG1MI was compared by means of TGA and DMTA tests. The plots are shown in Supplementary Materials Figures S1 and S2 in the supporting information. The use of BG1MI instead of 1MI does not affect the thermal stability of the material, and the TGA curves appear almost overlapped, with a 2% of weight loss at 269 °C for the material obtained with 1MI and at 271 °C for the other one. The peak of tan δ obtained by DMTA remains practically unchanged for both materials, with a shift in the temperature peak of only 2 °C higher in the case of the material prepared with 1MI. These results put in evidence that the use of the base generator derivative facilitates the manipulation of the initial formulation without any impact on the thermal characteristics of the material.

**Figure 1:**
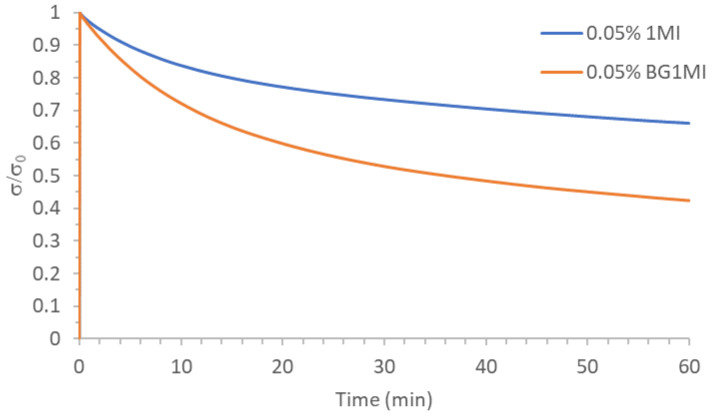
Normalized stress-relaxation behavior at 180 °C of the samples prepared with the same mol amount of 1MI and BG1MI.

The stress-relaxation curves obtained by DMTA of both PTUs are presented in Figure 1.

In the figure, we can see how the materials relax the stress at 180 °C, showing a quite slow relaxation process in both cases. However, it is evident that the use of BG1MI affects positively the relaxation rate if compared with 1MI. This effect is probably due to the presence of tetraphenylboronic acid released during the activation of the 1-methylimidazolium tetraphenylborate that acts as an additional catalyst. From this result, we can deduce that 1MI at this concentration is not an efficient catalyst to enhance the carbamate exchange process. Therefore, the effect on the stress-relaxation process of base generators prepared from DMAP, DBN, DBU and TBD, with higher values of pK_a_ and N, had to be tested.

**Figure 2:**
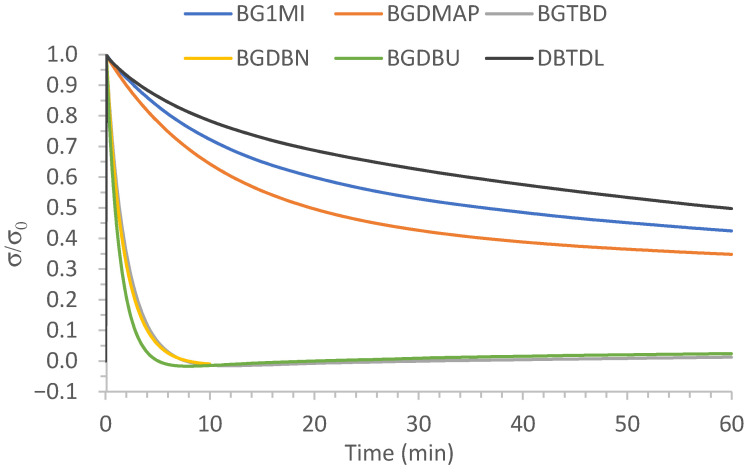
Normalized stress-relaxation plot at 180 °C of different poly(thiourethane) samples prepared with 0.05% mol of the different catalysts.

The relaxation rates of PTUs prepared with the different base generators were investigated at elevated temperatures (Figure 2), and the resulting relaxation behavior was compared with a PTU prepared by using DBTDL as the catalyst [6]. In all the BG samples, 0.05% mol of each tetraphenylborate salt was used as the catalyst.

The relaxation times (τ_0.37_), determined at the point when the stress decreased to 1/e (37%) of its initial value, are collected in Table 2. We can see in the figure how the mol proportion used in the case of DBTDL is not enough to obtain a fast relaxation process in the analyzed timescale; a higher amount of acid catalyst is needed to effectively catalyze the exchange reaction [5,6]. From the relaxation curves, it is clear how the base generators of strong bases and high nucleophilicity (DBN, DBU and TBD) accelerate the exchange mechanism, reaching τ_0.37_ in less than 2 min, in accordance with the conclusion reached by Bowman et al. [8], by using DBN as the catalyst. However, there is not a clear dependence of the relaxation time with the pK_a_ or N for the strong bases used, probably also due to the fact that the values of pK_a_ and N of each base are not too different from each other. Among all the BGs screened, we selected BGDBU as the catalyst to continue with further investigation, according to the fastest relaxation achieved with this system.

**Figure 3:**
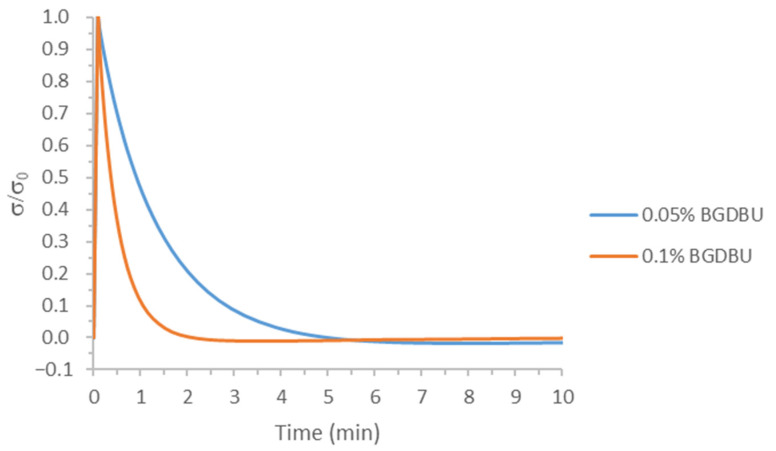
Normalized stress-relaxation plot at 180 °C of PTUs prepared with different mol proportions of BGDBU.

To see the effect of the proportion of BGDBU on the relaxation rate, we prepared a sample by doubling the amount of the catalyst (0.1% mol) in the formulation, proving that the rate of the exchange process increased significantly, as it can be seen in Figure 3. The τ_0.37_ at 180 °C is reached after only 30 s, confirming the positive influence of the catalyst concentration on the relaxation rate. A parallel behavior was already observed for the DBTDL catalyst in our previous paper, but with slower relaxation rates [6].

According to these results, we selected as the optimal catalyst molar proportion 0.1% to follow the study. Higher proportions of catalyst were not used, since the BG becomes difficult to dissolve in the thiol during the preparation of the formulation.

### 3.2. Study of the Effect of the Stoichiometry of the Formulation

The second main objective of this work consisted in the evaluation of the influence of the isocyanate/thiol ratio on the exchange mechanism and the relaxation behavior. The materials tested in the following sections were prepared to build evidence of the associative and/or dissociative trans-thiocarbamoylation exchange mechanism and their effect on the viscoelastic behavior. It was reported by Torkelson [5] and Bowman [8] that an associative exchange reaction occurs in the presence of an excess of thiol, while a dissociative exchange mechanism occurs when no excess of thiols is available. Both mechanisms can occur at the same time when stoichiometric thiol-isocyanate balance is maintained in the presence of a dual-role basic and nucleophilic catalyst. To be representative of the discussed cases, in addition to the thiol/isocyanate stoichiometric material, we prepared other two PTUs: (1) one with an excess of 10% in mol of HDI, with the assumption that this amount does not leave unreacted thiols in the thermosets, and (2) another with an excess of 10% in mol of thiol, to facilitate the associative pathway.

**Figure 4:**
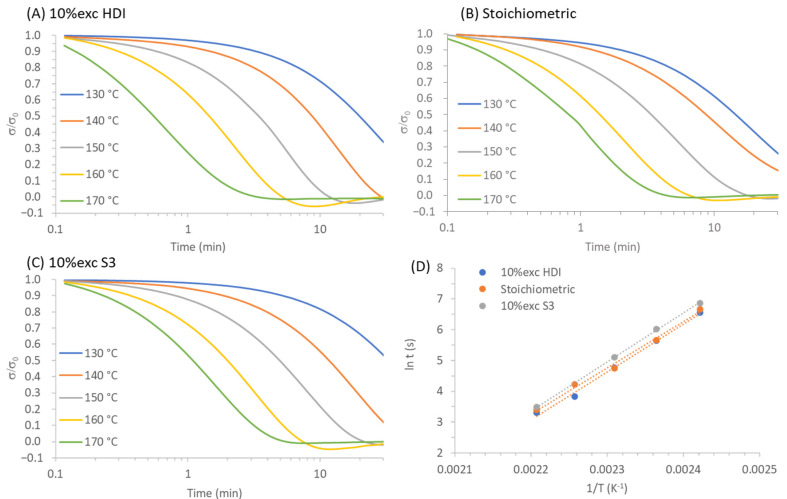
(**A**–**C**) Normalized stress-relaxation plots as a function of time at various temperatures from 130 to 170 °C during 30 min. (**D**) Arrhenius plot for the different poly(thiourethane) samples with 0.1% of BGDBU.

A series of stress-relaxation experiments were conducted on these three different systems, calculating their activation energy as it is represented in Figure 4.

As shown in Figure 4, a fast stress-relaxation was observed for all the systems at all temperatures analyzed. The relaxation times are very similar for the three PTUs prepared, and the characteristic time of relaxation decreased from approximately 30 to 1 min as the temperature increased from 130 to 170 °C. The highest relaxation rate was obtained for the system with an excess of isocyanate.

The logarithm of the relaxation times was plotted as a function of the inverse of temperature for all the materials prepared, fitting perfectly with an Arrhenius-like behavior. The activation energies and the topology freezing temperatures were calculated by the Arrhenius equation, using the slope of the straight line. The values obtained are collected in Table 3. As it can be appreciated, the activation energies are similar for the three PTUs studied, around 120–130 kJ/mol. Moreover, from the stress-relaxation experiments, no significant differences in the behavior are appreciable that can lead us to consider a clear change in the trans-thiocarbamoylation pathways by changing the stoichiometry of the formulation in the selected range. It can be observed that the materials prepared with an excess of thiol, which should enhance the associative exchange rate, are the slowest materials in the relaxation process. This behavior seems to suggest that dissociative mechanism is predominant with the catalyst used.

### 3.3. Study of the Thermal Stability of the PTUs Prepared

**Figure 5:**
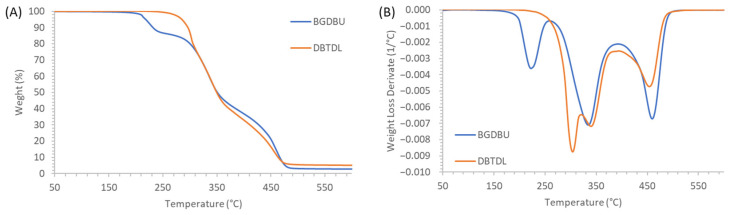
(**A**) Thermogravimetric analysis (TGA) curves and (**B**) DTG curves of the poly(thiourethane)s prepared with 0.1% of BGDBU and 1% of DBTDL.

The thermal stability of the PTUs prepared by using BGDBU as the catalyst was evaluated by TGA and compared with the material prepared from DBTDL, since it is important to prove that, at the reshaping temperature, the material does not lose any volatile product. Moreover, the study of the thermal degradation mechanism could help to disclose about the adoption of a dissociative interchange mechanism. The TGA and the derivative of the loss weight curve (DTG) curves are shown in Figure 5.

As we can see from Figure 5, the thermal stability of the material based on BGDBU decreases if compared with the PTU prepared with the acidic catalyst, DBTDL.

The DTG plot shows three clear degradation steps for both materials. The maximum of the first degradation peak appears at 305 °C for the DBTDL sample, whereas, in the case of BGDBU samples, it is shifted to a lower temperature (around 220 °C) and becomes well separated from the second one. Since the first degradation step was attributed, in several studies on PU [36], to the reversion of urethanes to isocyanate and alcohols, it can be easy to make the assumption that an analogous degradation to isocyanate and thiols could occur in PTU networks [37]. It should be noticed that, in a previous study, we could not see great differences between the materials prepared with DBTDL and those prepared by using BG1MI as the catalyst [19]. This means that DBU has a main role in the thermal degradation of the PTUs prepared with BGDBU, which could be attributed to its higher nucleophilic character, facilitating a dissociative mechanism.

**Figure 6:**
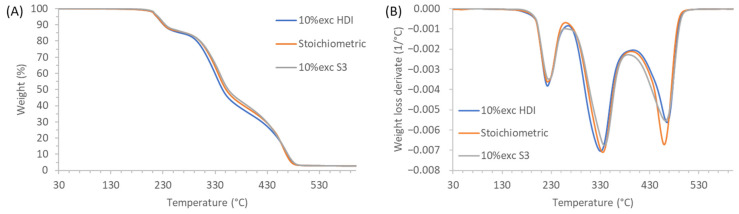
TGA curves (**A**) and DTG curves (**B**) of the poly(thiourethane)s prepared from different stoichiometric formulations with 0.1% of BGDBU.

By analyzing the TGA curves of the PTUs prepared with different thiol-isocyanate stoichiometric ratio (see Figure 6) and in presence of BGDBU, it can be observed that the 2% of loss in weight (given in Table 4) occurs at temperatures around 210 °C for all the samples, without significant differences among them. DTG curves are very similar for all the materials and show the three decomposition processes. The first degradation step, at around 220 °C, relates to a 15% of mass loss; the second peak, at 340 °C, is related to a mass loss of an additional 45%; and the third step is associated with a complete degradation of the network. No differences in the thermal degradation are appreciable on changing the stoichiometry of one of the monomers used in the preparation of the material, and this could be related to the similar behavior observed in the relaxation experiments of the three samples.

The good separation between the three different processes has driven us to perform a kinetic analysis of the degradation process. Thus, we carried out an isoconversional analysis of the three degradation steps, to calculate the activation energy and the kinetic parameters which best describe the degradation processes of PTUs. To this aim, a series of TGA dynamic experiments were performed at different heating rates, as shown in Supplementary Materials Figure S3, for the stoichiometric PTU. As expected, as the heating rate increases, the TGA and DTG curves shift to higher temperatures.

From the dynamic isoconversional kinetic study, we calculated the activation energy for the three different materials, using the Equation (5) applied to the degradation process in N_2_ atmosphere. Since isoconversional activation energies are relatively constant during each degradation step, their average values are reported in Table 4.

The kinetic of each degradation step was studied, considering in Equation (2) as final mass (m_∞_) the mass of the remaining material at the end of this stage. The *E_a_* during the first degradation step, calculated as described in the Section 3, remains relatively constant implying that a single degradation mechanism occurs in the considered range of temperatures. The *E_a_* calculated is around 130 kJ/mol. This value of *E_a_* is comparable for all the different PTUs analyzed, and its minimum value is observed for the material prepared with an excess of isocyanate.

For a better understanding of the processes that occur during the decomposition of the PTU, FTIR analyses were performed on the partially degraded material after step 1 and step 2 of degradation. The material was heated up in the TGA until finishing each degradation step, and then the partially degraded samples were analyzed by FTIR-ATR at room temperature. In Supplementary Materials Figure S4, the FTIR of the initial and the degraded material until 260 °C are presented. From the spectra it is possible to observe how the characteristic carbonyl band of thiourethane at 1670 cm^−1^ is almost completely disappeared during the first degradation step demonstrating the decomposition of the thiourethane group. However, any absorption of isocyanate at 2250 cm^−1^ could be detected, such as reported by Rogulska et al. [37], who observed a small peak, attributable to isocyanate formation during degradation, although in that case DBTDL was used as the catalyst. To analyze the second degradative process, the material was heated up in the TGA until 340 °C and the FTIR spectra was collected (presented in Supplementary Materials Figure S5). By analyzing the spectra, we can state that the second degradation step is associated with the decomposition of the ester bond of the thiol structural units, as evidenced by the drastic decrease of the peak at 1710 cm^−1^.

Additionally, the volatiles produced during the first decomposition step were studied by gas chromatography coupled to a mass spectrometer detector (GC-MS), following the procedure described in the Section 2.8. Supplementary Materials Figure S6 illustrates the chromatographic profile of volatile compounds obtained by GC-MS, where two main peaks were detected and identified by mass spectroscopy (Supplementary Materials Figures S7 and S8). The peak at 2.28 min is due to the formation of benzene in the degradation, and it can be associated to the aromatic ring of the tetraphenylboronic acid. The peak at 1.21 min is due to the formation of carbonyl sulfide, which indicates that, at high temperatures, the thiourethane moiety is decomposed mainly through a CSO elimination, which is the responsible of the initial weight loss. This is in accordance with the results of the degradation study previously reported by Rogulska et al. [37], where the elimination of CSO and formation of an amine was found to be the main responsible for the degradation of poly(thiourethane) polymers. It must be pointed out that CSO can only be formed directly from the thiourethane moieties and not after scission of this group. No free diisocyanate was detected by GC-MS, which may indicate that the reversion of thiourethane linkages to isocyanate and thiol is very fast and reversible, and no loss of diisocyanates or thiols was detected. This observation fits with the results of our previous studies [6] in which, during the interchange mechanism of a model compound, no isocyanate or free thiols were detected. Thus, associative and dissociative trans-thiocarbamoylation mechanisms behave similarly in terms of average crosslinking density during the topological changes, and therefore any sudden decrease of the viscosity during the reshaping processes is produced.

**Figure 7:**
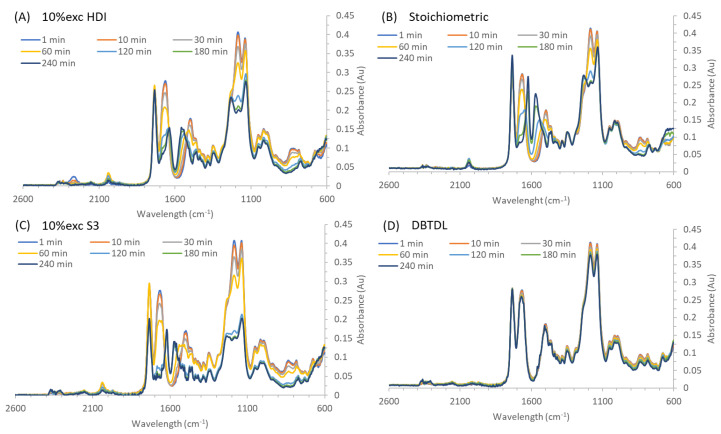
FTIR of the different poly(thiourethane) samples with 0.1% of BGDBU and 1% of DBTDL registered during 4 h at 180 °C.

Although no significant differences in weight loss were determined by TGA on changing the stoichiometry of the material, FTIR-ATR was used to analyze structural effects of a change in stoichiometric when heating the samples at 180 °C during 4 h. We have selected this temperature to detect the occurrence of a possible dissociation process of poly(thiourethane) in isocyanate and thiol groups, which could be evidenced by the appearance of an isocyanate absorption peak. For comparison purposes, we also analyzed a PTU sample obtained with DBTDL. The spectra registered during the thermal treatment are shown in Figure 7.

It is possible to see in Figure 7 that the starting spectra of the samples prepared with BGDBU or DBTDL are identical, and only in the initial spectra of the PTU with an excess of isocyanate can be appreciated the typical absorption peak at 2270 cm^−1^, corresponding to the unreacted isocyanate. During the heat treatment at 180 °C for 4 h, it was possible to observe the degradation processes and that the intensity of the different peaks was reduced. However, isocyanate absorption was not detected in any case. The typical carbonyl band of thiourethane at 1670 cm^−1^ was almost drastically reduced after the heating process, due to the degradation of these moieties. Instead, in presence of 1% of DBTDL in stoichiometric formulation, no appreciable evidence of degradative process was detected, confirming that thiourethane groups were not decomposed, as already shown in our previous work [6]. From this thermal study, we can conclude that the use of base generators derived from high nucleophilic bases, like DBU, is quite convenient for a fast reshaping and relaxation-stress purposes, but too high temperatures or too long processing times should be avoided, since both can lead to a decomposition of the materials that worsens the mechanical performance.

### 3.4. Study of the Recycling Process

To investigate the recyclability of the three different crosslinked PTUs prepared, the materials were cut into small pieces and hot-pressed at 15 MPa, in an aluminum mold. The temperature of the recycling process was selected according to the isothermal degradation times estimated by using Equation (5) and the isoconversional non-isothermal kinetic parameters previously determined. Thus, 130 °C was chosen as the temperature that combines a fast relaxation process, together with a high thermal stability, since, by the data obtained at 130 °C, a weight loss of 1% is estimated to be above 9 h.

**Figure 8:**
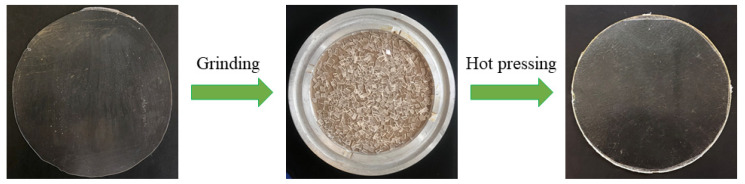
Photographs of the original, grinded and recycled sample prepared from stoichiometric PTU with a 0.1% of BGDBU.

Figure 8 shows the pictures taken from the original, grinded and reprocessed samples prepared as described in the experimental part. As we can see, the original and the recycled materials both show good transparency. It can be also appreciated the excellent uniformity of the recycled sample.

To evaluate the recyclability of the PTUs prepared, the original and the recycled samples were subjected to uniaxial tensile test, DMTA and FTIR analysis. Dog-bone-shaped samples of the original and recycled PTUs were tested at room temperature, until break, and their stress-strain behavior was analyzed, obtaining the tensile modulus and the yield stress and strain. These mechanical parameters, as well as thermomechanical data obtained, are collected in Table 5.

From the tensile tests, it can be observed that the mechanical properties of the PTUs after the first recycling process are very similar to those of the original samples for all the systems studied. The stoichiometric material, as expected, is the most rigid at room temperature, showing the highest values of tensile modulus and yield stress, in the original and recycled samples. Moreover, the recycled sample of the stoichiometric material presents excellent mechanical performance in comparison with the original one, i.e., a tensile modulus around 85% of the original modulus and a yield stress of almost 96%. The materials prepared out of stoichiometry also showed a very similar mechanical behavior, with a tensile modulus of 1.7 and 1.8 GPa for the material prepared with an excess of isocyanate and thiol, respectively. The mechanical properties of the recycled samples in these two materials are perfectly recovered, with values that fall within the range of the experimental error.

**Figure 9:**
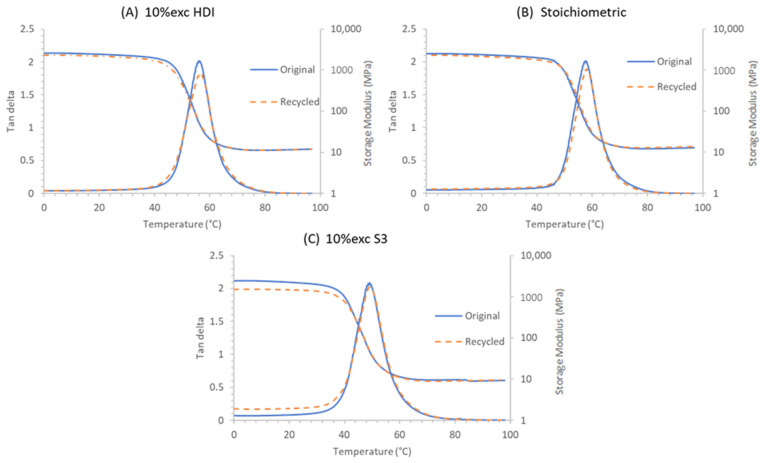
Dependence of tan δ and storage modulus versus temperature of the different materials prepared before and after recycling.

To compare the thermomechanical behavior of the original and recycled samples, the variation of the storage modulus and the tan δ curves with the temperature extracted from DMTA analysis are presented in Figure 9, and the most representative data are collected in Table 5. In the figure, it can be appreciated that the thermomechanical behavior of the original and recycled materials is very similar, with both the shape and position of tan δ curves remaining unaltered. From these results, we can assert that these thermosets can be recycled without significant difference in the thermomechanical properties, showing the promising use of BGDBU as an efficient catalyst to the trans-thiocarbamoylation exchange of poly(thiourethane)s.

**Figure 10:**
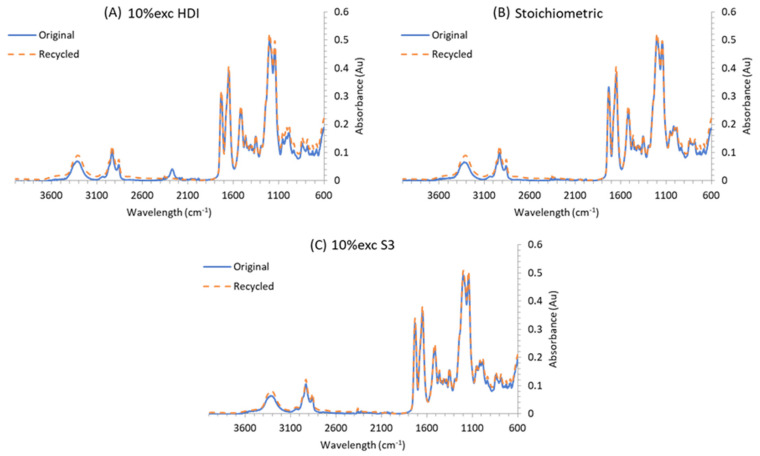
FTIR of poly(thiourethane)s before and after recycling registered at room temperature.

To confirm that no changes in the chemical structure of the materials have occurred after the recycling process, the FTIR spectra of the original and recycled materials were recorded and overlapped (see Figure 10). The FTIR spectra of the PTUs prepared remain almost identical after the recycling process. The only appreciable changes are detected in the material prepared with an excess of isocyanate, where it is possible to see how the absorption band of the isocyanate cannot be detected in the recycled material. Thus, we can confirm the recyclability of this class of thermosets, even in different stoichiometric ratios, in the presence of a strong basic and nucleophilic catalyst, as reported by Bowman [8] for DBN catalyst in elastomeric PTUs.

### 3.5. Dissolution Experiments

Typical thermosets do not solubilize in any type of solvent. However, covalent adaptable networks can be solubilized by a suitable solvent when their chemical-exchange process proceeds through a dissociative mechanism. The possibility to undergo complete dissolution of these reprocessable thermosets could constitute an alternative chemical recycling route to the mechanical recycling previously studied.

Hillmeyer et al. [14] recently reported that poly(urethane) thermosets can be dissolved in dimethyl sulfoxide (DMSO), thus demonstrating the dissociative nature of the urethane exchange pathway; however, the same sample was not soluble in a chlorinated aromatic solvent. This was attributed to the fact that the sample had increased swellability in DMSO because of the higher polarity. In our study, we tried to dissolve the PTU polymer in DMSO and dichlorobenzene (DCB). After leaving the PTUs for 24 h at 150 °C in both solvents, we noticed that the sample in DCB had swollen but had not dissolves, and once the solvent was completely removed the soluble fraction, it was only 4%. However, the sample immersed in DMSO was almost completely dissolved after 24 h, as we can observe in Supplementary Materials Figure S9. The dissolution of the sample in DMSO confirms that the dissociative exchange mechanism plays a role in the reconfiguration of the network. In fact, when a good solvent, such as DMSO, is used, the broken chains remain in solution without reverting to crosslinked PTU networks, which would be insoluble.

## 4. Conclusions

In this work, the catalytic effectiveness of tetraphenylborate derivatives of DBN, DBU and TBD in the trans-thiocarbamoylation process in poly(thiourethane) thermosets was demonstrated. The relaxation rate of the PTUs prepared with the abovementioned catalysts is faster than the one prepared with DBTDL. The great catalytic effect is due not only to the high basicity and nucleophilicity of the amines, but also to the presence of the tetraphenylboric acid, which is formed when the amine is thermally released.

Moreover, the use of the tetraphenylborate salts of these amines is advantageous in comparison to the use of the free amines, concerning the manipulation of the formulation: It helps to delay the pot-life of the mixture and gives the possibility to increase the amount of catalyst in the formulation without using any solvent, increasing thus the exchange rate of the covalent bond.

The poly(thiourethane)s networks show, in the presence of these new catalysts, a vitrimer-like behavior with the possibility to be reshaped and recycled. The materials obtained degrade at temperatures lower than those obtained with DBTDL as the catalyst; therefore, the recycling parameters, temperature and time were properly selected by a thermal degradation study, demonstrating that, under adequate recycling conditions, the materials keep their good thermal and mechanical performance and their chemical structure. Materials with a 10% of molar stoichiometric imbalance, in isocyanate or thiol groups, do not show appreciable differences in relaxation rate and recycling capability.

Moreover, it has been demonstrated that the thermal degradation leads to an initial loss of carbonyl sulfide without dissociation of thiourethane moieties to isocyanate and thiol groups. This observation, together with the proved solubility of the material in DMSO and the unaffected relaxation rate in the presence of an excess of thiol, confirms that the exchange mechanism is mainly dissociative, but with a very fast equilibrium process that leads to a vitrimer-like behavior.

## Figures and Tables

**Table 1:** Formulations used to prepare poly(thiourethane) thermosets.

Formulation	Diisocyanate (g)	Thiol (g)	BGDBU 0.1% (mg)
10% exc HDI	2.09	3.00	9.30
Stochiometric	1.90	3.00	9.30
10% exc S3	1.90	3.30	9.30

**Table 2:** Characteristic relaxation time in stress-relaxation experiments for poly(thiourethane) (PTU) samples with a 0.05% in mol of the different catalysts used.

Samples	τ_0.37_ (min) ^1^
BG1MI	Not reached in 1 h
BGDMAP	45
BGTBD	1.7
BGDBN	1.7
BGDBU	1.3
DBTDL	Not reached in 1 h

^1^ Time to reach a value of σ/σ_0_ = 0.37 at 180 °C.

**Table 3:** Activation energy, topology freezing temperature and time for reach a value of σ/σ_0_ = 0.37 from the stress-relaxation experiments at different temperatures.

Samples	*Ε_a_* (kJ/mol)	*T_v_* (°C)	*τ*_0.37−130 °C_ (min)	*τ*_0.37−140 °C_ (min)	*τ*_0.37−150 °C_ (min)	*τ*_0.37−160 °C_ (min)	*τ*_0.37−170 °C_ (min)
10% exc HDI	132	94	27.8	11.7	4.7	1.9	0.8
Stoichiometric	117	87	21.5	13.0	4.8	1.9	1.1
10% exc S3	127	96	45.2	16.0	6.9	2.7	1.5

**Table 4:** Temperature of initial degradation and activation energies of the degradation steps of the PTUs prepared with 0.1% of BGDBU.

Samples	T_2%_ (°C)	Ε_a I peak_ (kJ/mol)	Ε_a II peak_ (kJ/mol)	Ε_a III peak_ (kJ/mol)
10% exc HDI	209	127	170	257
Stoichiometric	209	130	158	275
10% exc S3	212	139	149	259

**Table 5:** Mechanical and thermomechanical results of the original and recycled PTUs materials tested. The average value of the results for three different samples tested is presented. Coefficients of variations are less than 7% for stress-and-strain results and less than 5% for the tensile moduli.

Original						
Samples	*E*^1^ (GPa)	*σ_max_*^2^ (MPa)	*ε_max_*^3^ (%)	*T_tanδ_*^4^ (°C)	FWHM ^5^ (°C)	*E*’ ^6^ (MPa)
10% exc HDI	1.7	30.4	3.0	56.3	9.6	11.4
Stoichiometric	2.5	45.1	2.5	57.4	9.9	12.7
10% exc S3	1.8	31.4	2.7	49.0	10.8	9.6
**Recycled**						
**Samples**	***E*** **^1^** **(GPa)**	***σ_max_*** **^2^** **(MPa)**	***κ*** ***_max_*** **^3^** **(%)**	***T_tanδ_*^4^** **(°C)**	**FWHM ^5^** **(°C)**	***E*** **’ ^6^** **(MPa)**
10% exc HDI	1.6	28.9	3.1	56.3	10.7	11.3
Stoichiometric	2.1	43.3	2.8	57.5	9.2	13.0
10% exc S3	1.8	32.2	2.5	49.2	10.9	9.2

^1^ Tensile modulus at room temperature. ^2^ Yield Stress. ^3^ Yield Strain. ^4^ Temperature of the maximum of the tan δ peak. ^5^ Full width at half maximum of the tan δ peak. ^6^ Relaxed modulus determined at the temperature of the maximum of tan δ peak +30 °C.

## Supplementary Materials

The following are available online at https://www.mdpi.com/2073-4360/12/12/2913/s1. Figure S1: TGA curves of the samples prepared with the same mol proportion of 1MI and BG1MI. Figure S2: Evolution of tan δ of the PTU samples prepared with the same mol proportion of 1MI and BG1MI. Figure S3: (A) TGA and (B) DTG curves of the poly(thiourethane)s prepared in stoichiometric ratio with 0.1% of BGDBU at different heating rates. Figure S4: FTIR of stoichiometric poly(thiourethane), before and after heating up the material until 260 °C in the TGA at 10 °C/min. Figure S5: FTIR of stoichiometric poly(thiourethane), after heating up the material until 340 °C in the TGA at 10 °C/min. Figure S6: Gas chromatograms of the sample after heating a stoichiometric PTU sample for 1 h at 200 °C. Figure S7: Mass spectrum of the eluted product at 1.84 min. Figure S8: Mass spectrum of the eluted product at 1.24 min. Figure S9: Dissolution experiment at 150 °C in dimethyl sulfoxide (DMSO) and dichlorobenzene (DCB).

## References

[1] Scheutz G.M., Lessard J.J., Sims M.B., Sumerlin B.S. (2019). Adaptable Crosslinks in Polymeric Materials: Resolving the Intersection of Thermoplastics and Thermosets. J. Am. Chem. Soc..

[2] Podgórski M., Fairbanks B.D., Kirkpatrick B.E., McBride M., Martinez A., Dobson A., Bongiardina N.J., Bowman C.N. (2020). Toward Stimuli-Responsive Dynamic Thermosets through Continuous Development and Improvements in Covalent Adaptable Networks (CANs). Adv. Mater..

[3] Guerre M., Taplan C., Winne J.M., Du Prez F.E. (2020). Vitrimers: Directing chemical reactivity to control material properties. Chem. Sci..

[4] Elling B.R., Dichtel W.R. (2020). Reprocessable Cross-Linked Polymer Networks: Are Associative Exchange Mechanisms Desirable?. ACS Cent. Sci..

[5] Li L., Chen X., Torkelson J.M. (2019). Reprocessable polymer networks via thiourethane dynamic chemistry: Recovery of cross-link density after recycling and proof of principle solvolysis leading to monomer recovery. Macromolecules.

[6] Gamardella F., Guerrero F., De la Flor S., Ramis X., Serra A. (2020). A new class of vitrimers based on aliphatic poly(thiourethane) networks with shape memory and permanent shape reconfiguration. Eur. Polym. J..

[7] Gamardella F., De la Flor S., Ramis X., Serra A. (2020). Recyclable poly(thiourethane) vitrimers with high Tg. Influence of the isocyanate structure. React. Funct. Polym..

[8] Wen Z., Han X., Fairbanks B.D., Yang K., Bowman C.N. (2020). Development of thiourethanes as robust, reprocessable networks. Polymer.

[9] Ireni N.G., Narayan R., Basak P., Raju K.V.S.N. (2016). Poly(thiourethane-urethane-urea) as anticorrosion coatings with impressive optical properties. Polymer.

[10] Jaffrennou B., Droger N., Mechin F., Halary J.L., Pascault J.P. (2005). Characterization structural transitions and properties of a tightly crosslinked polythiourethane network for optical applications. e-Polymers.

[11] Zheng N., Fang Z., Zou W., Zhao Q., Xie T. (2016). Thermoset shape-memory polyurethane with intrinsic plasticity enabled by transcarbamoylation. Angew. Chem..

[12] Chen X., Li L., Jin K., Torkelson J.M. (2017). Reprocessable polyhydroxyurethane networks exhibiting full property recovery and concurrent associative and dissociative dynamic chemistry via transcarbamoylation and reversible cyclic carbonate aminolysis. Polym. Chem..

[13] Fortman D.J., Sheppard D.T., Dichtel W.R. (2019). Reprocessing cross-linked polyurethanes by catalyzing carbamate exchange. Macromolecules.

[14] Brutman J.P., Fortman D.J., De Hoe G.X., Dichtel W.R., Hillmyer M.A. (2019). Mechanistic study of stress relaxation in urethane-containing polymer networks. J. Phys. Chem. B.

[15] Kultys A., Rogulska M., Pikus S. (2008). The synthesis and characterization of new thermoplastic poly (thiourethane−urethane)s. J. Polym. Sci. Part A Polym. Chem..

[16] Lu C., Guan C., Liu Y., Cheng Y., Yang B. (2005). PbS/Polymer nanocomposite optical materials with high refractive index. Chem. Mater..

[17] Chandrasekaran S. (2016). Click Reactions in Organic Synthesis.

[18] Shin J., Matsushima H., Comer C.M., Bowman C.N., Hoyle C.E. (2010). Thiol-isocyanate-ene ternary networks by sequential and simultaneous thiol click reactions. Chem. Mater..

[19] Gamardella F., Ramis X., De la Flor S., Serra A. (2019). Preparation of poly(thiourethane) thermosets by controlled thiol-isocyanate click reaction using a latent organocatalyst. React. Funct. Polym..

[20] Oliveira V.D.G., Cardoso M.F.D.C., Forezi L.D.S.M. (2018). Organocatalysis: A Brief Overview on Its Evolution and Applications. Catalysts.

[21] Sardon H., Pascual A., Mecerreyes D., Taton D., Cramail H., Hedrick J.L. (2015). Synthesis of polyurethanes using organocatalysis: A perspective. Macromolecules.

[22] Van Zee N.J., Nicolaÿ R. (2020). Vitrimers: Permanently crosslinked polymers with dynamic network topology. Prog. Polym. Sci..

[23] Denissen W., Droesbeke M., Nicolaÿ R., Leibler L., Winne J.M., Du Prez F.E. (2017). Chemical control of the viscoelastic properties of vinylogous urethane vitrimers. Nat. Commun..

[24] Huang S., Podgórski M., Han X., Bowman C.N. (2020). Chemical recycling of poly(thiourethane) thermosets enabled by dynamic thiourethane bonds. Polym. Chem..

[25] Maji B., Stephenson D.S., Mayr H. (2012). Guanidines: Highly Nucleophilic Organocatalysts. ChemCatChem.

[26] Baidya M., Mayr H. (2008). Nucleophilicities and carbon basicities of DBU and DBN. Chem. Commun..

[27] Rodima T., Kaljurand I., Pihl A., Mäemets V., Leito I., Koppel I.A. (2002). Acid-base equilibria in nonpolar media. 2.1 Self-consistent basicity scale in THF solution ranging from 2-methoxypyridine to EtP1(pyrr) phosphazene. J. Org. Chem..

[28] Konuray O., Fernández-Francos X., Ramis X. (2017). Latent curing of epoxy-thiol thermosets. Polymer.

[29] Konuray O., Liendo F., Fernández-Francos X., Serra À., Sangermano M., Ramis X. (2017). Sequential curing of thiol-acetoacetate-acrylate thermosets by latent Michael addition reactions. Polymer.

[30] Sun X., Gao J.P., Wang Z.Y. (2008). Bicyclic guanidinium tetraphenylborate: A photobase Generator and A Photocatalyst for living anionic ring-opening polymerization and cross-linking of polymeric materials containing ester and hydroxy groups. J. Am. Chem. Soc..

[31] Vyazovkin S. (2015). Isoconversional Kinetics of Thermally Stimulated Processes.

[32] Vyazovkin S., Sbirrazzuoli N. (1999). Kinetic methods to study isothermal and nonisothermal epoxy-anhydride cure. Macromol. Chem. Phys..

[33] Coats A.W., Redfern J.P. (1964). Kinetic parameters from thermogravimetric data. Nature.

[34] Blaine R.L., Kissinger H.E. (2012). Homer Kissinger and the Kissinger equation. Thermochim. Acta.

[35] Ramis X., Cadenato A., Salla J.M., Morancho J.M., Vallés A., Contat L., Ribes A. (2004). Thermal degradation of polypropylene/starch-based materials with enhanced biodegradability. Polym. Degrad. Stab..

[36] Delebecq E., Pascault J.P., Boutevin B., Ganachaud F. (2012). On the versatility of urethane/urea bonds: Reversibility, blocked isocyanate, and non-isocyanate polyurethanes. Chem. Rev..

[37] Rogulska M., Kultys A., Olszewska E. (2013). New thermoplastics poly(thiourethane-urethane) elastomers based on hexane-1,6-diyl diisocyanate (HDI). J. Therm. Anal. Calorim..

