# Supplementary material for: Recyclable Organocatalyzed Poly(Thiourethane) Covalent Adaptable Networks
Source: Polymers (Basel). 2020 Dec 4;12(12):2913. doi: 10.3390/polym12122913 (PMC7761908; doi:10.3390/polym12122913)
Supplement: Supplementary file 1 [file polymers-12-02913-s001.pdf]

SUPPORTING INFORMATION

RECYCLABLE ORGANOCATALYZED POLY(THIOURETHANE) COVALENT ADAPTABLE NETWORKS

Francesco Gamardella,[1] Sara Muñoz,[2] Silvia De la Flor,[2] Xavier Ramis[3] and Angels Serra[1*]

a. [1] Dept. of Analytical and Organic Chemistry, Universitat Rovira i Virgili, C/ Marcel·lí Domingo 1, Building. N4. 43007, Tarragona, Spain.; e-mail@e-mail.com

b. [2] Dept. of Mechanical Engineering, Universitat Rovira i Virgili, Av. Països Catalans, 26, 43007 Tarragona, Spain.

c. [3] Thermodynamics Laboratory, ETSEIB Universitat Politècnica de Catalunya, Av. Diagonal, 08028, Barcelona, Spain.

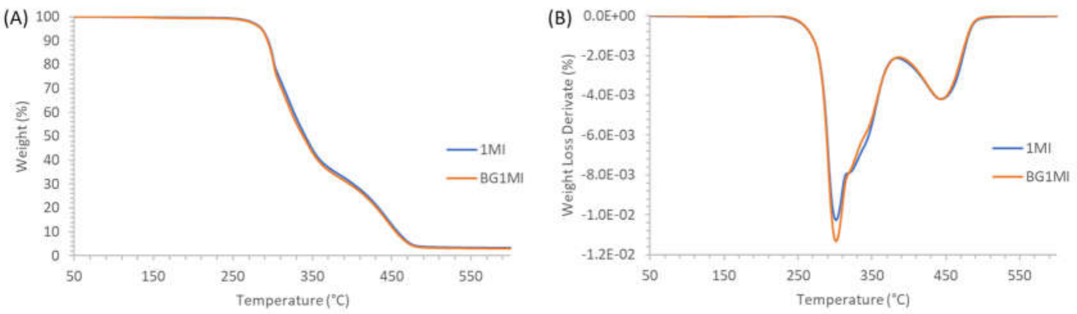

Figure S1. TGA curves of the samples prepared with the same mol proportion of 1MI and BG1MI (A). Curves of the rate of weight loss against temperature, DTG (B).

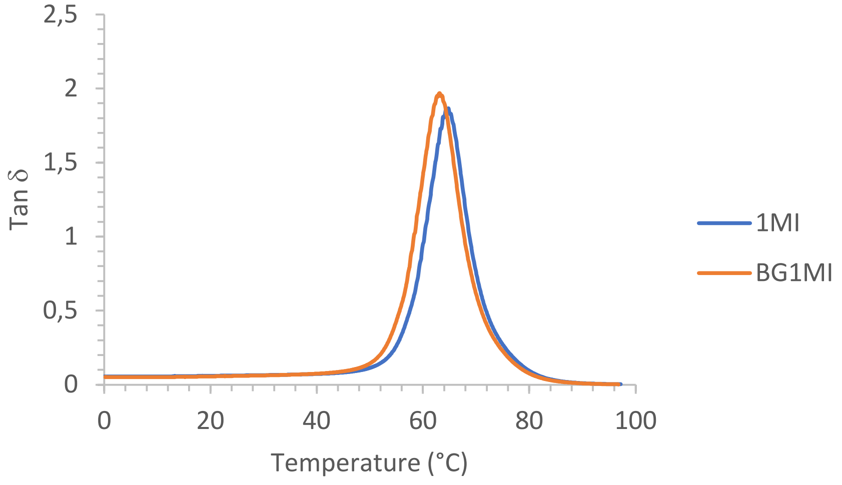

Figure S2. Evolution of tan δ against temperature of the PTU samples prepared with the same mol proportion of 1MI and BG1MI

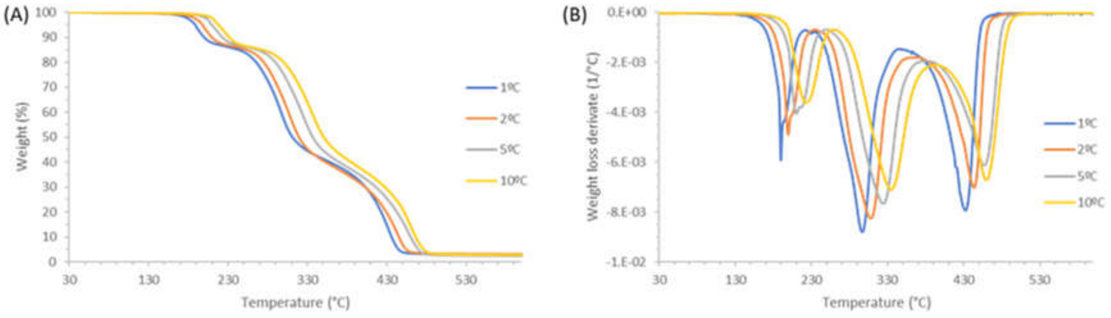

Figure S3. (A) TGA and (B) DTG curves of the poly(thiourethane)s prepared in stoichiometric ratio with 0.1% of BGDBU as the catalyst at different heating rates.

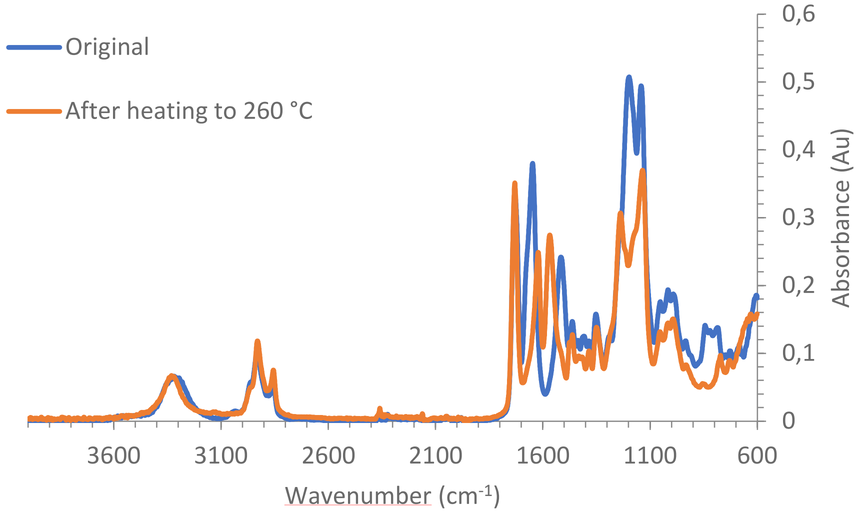

Figure S4. FTIR of stoichiometric poly(thiourethane), registered at room temperature, before and after heating up the material until 260 °C in the TGA at 10 °C/min.

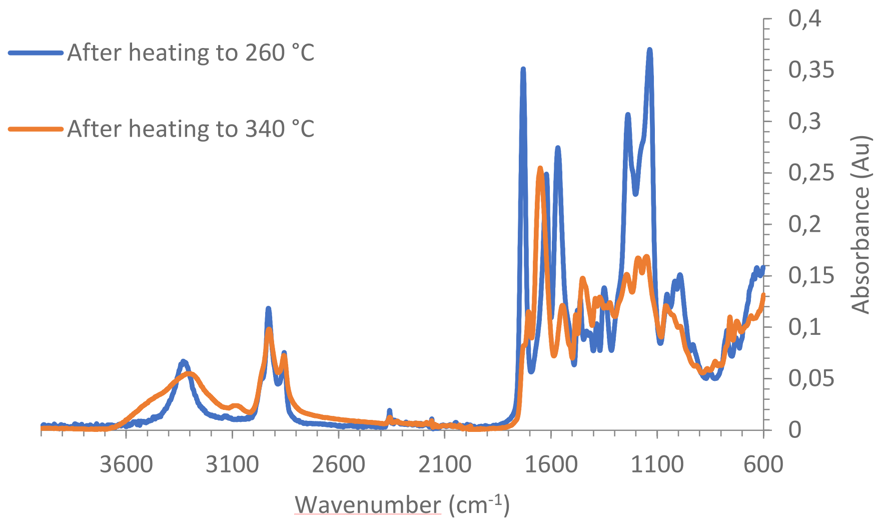

Figure S5. FTIR of stoichiometric poly(thiourethane), registered at room temperature, after heating up the material until 340 °C in the TGA at 10 °C/min.

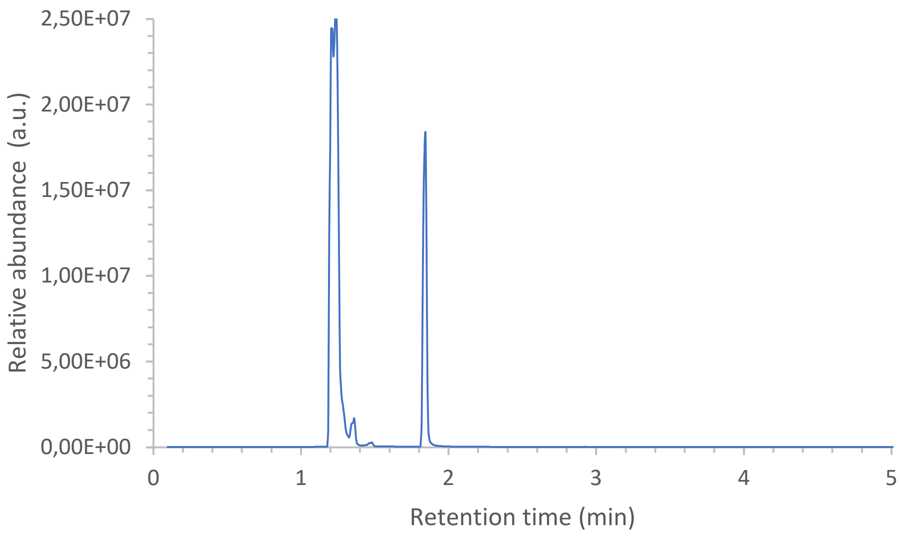

Figure S6. Gas-chromatograms of the sample after heating a stoichiometric PTU sample for 1h at 200 °C.

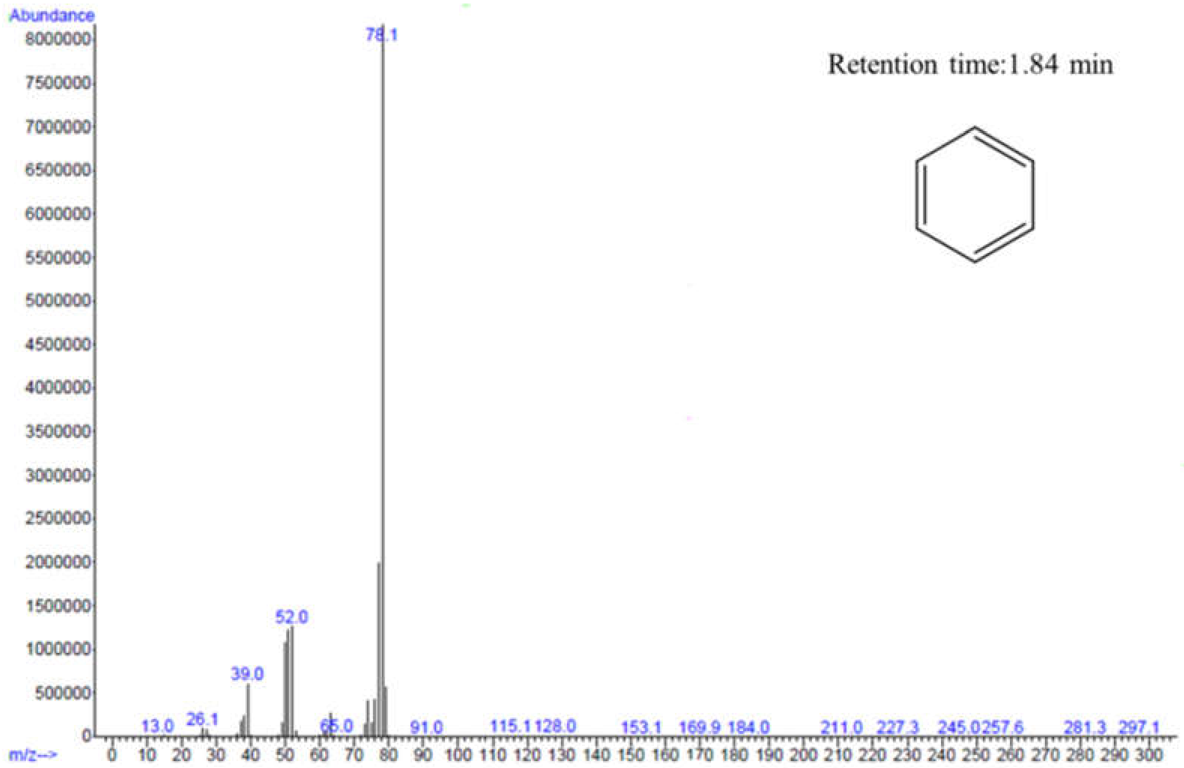

Figure S7. Mass spectrum of the eluted product at 1.84 min, identified as benzene, that corresponds to the decomposition of the sodium tetraphenylborate moiety.

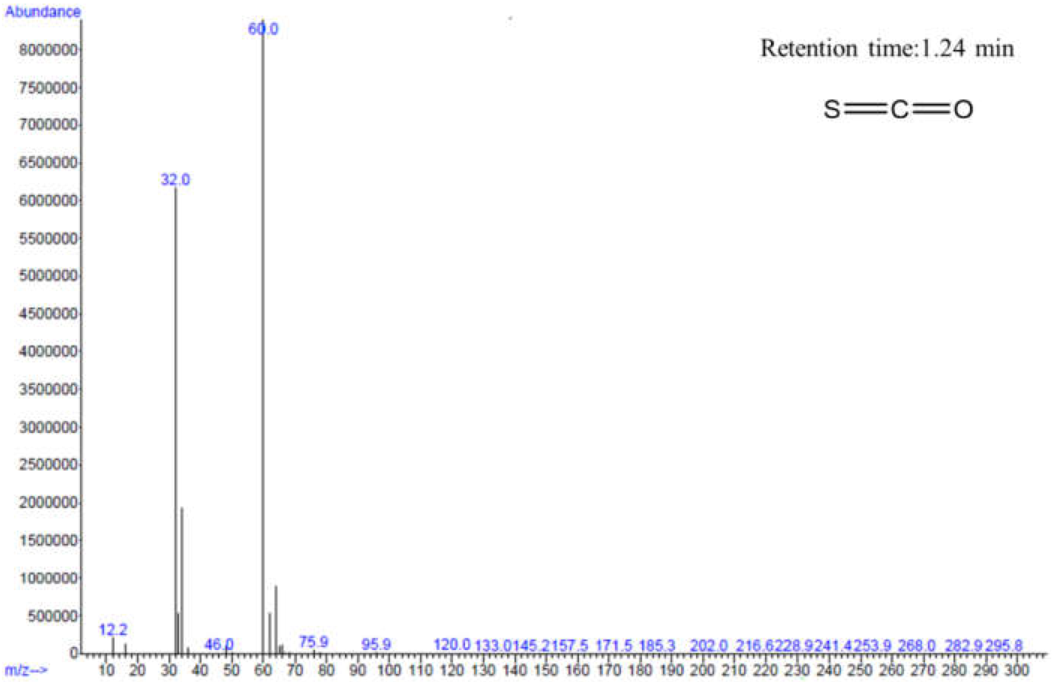

Figure S8. Mass spectrum of the eluted product at 1.24 min that corresponds to the carbonyl sulphide.

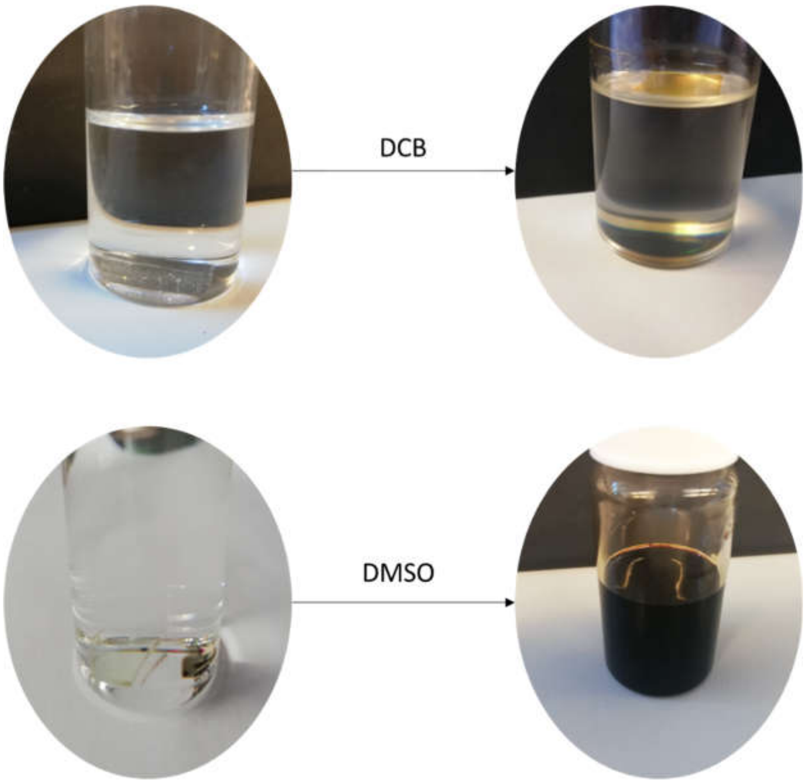

Figure S9. Dissolution experiment at 150 °C in dimethyl sulfoxide (DMSO) and dichlorobenzene (DCB). The sample is at the bottom of the vial in DMSO but floats in DCB, due to the different density of the solvent.
